# Arbutin attenuates behavioral impairment and oxidative stress in an animal model of Parkinson's disease

**Published:** 2018

**Authors:** Masoumeh Dadgar, Mahdi Pouramir, Zohreh Dastan, Maryam Ghasemi-Kasman, Manouchehr Ashrafpour, Ali Akbar Moghadamnia, Soraya Khafri, Mohsen Pourghasem

**Affiliations:** 1 *Student Research Committee, Babol University of Medical Sciences, Babol, Iran*; 2 *Department of Clinical Biochemistry, Faculty of Medicine, Babol University of Medical Sciences, Babol, Iran*; 3 *Cellular and Molecular Biology Research Center, Health Research Institute, Babol University of Medical Sciences, Babol, Iran *; 4 *Neuroscience Research Center, Health Research Institute, Babol University of Medical Sciences, Babol, Iran *; 5 *Department of Physiology, Faculty of Medicine, Babol University of Medical Sciences, Babol, Iran*; 6 *Department of Pharmacology, Faculty of Medicine, Babol University of Medical Sciences, Babol, Iran*; 7 *Department of Biostatistics and Epidemiology, Babol University of Medical Sciences, Babol, Iran*; 8 *Department of Anatomy, Embryology and Histology, Faculty of Medicine, Babol University of Medical Sciences, Babol, Iran*

**Keywords:** Arbutin, Parkinson’s disease, Behavioral impairment, Oxidative stress, Nitrosative stress

## Abstract

**Objective:**

Arbutin has been shown to have antioxidant and free-radical scavenging properties. The aim of this study was to investigate the effects of arbutin administration on behavioral impairment, and oxidative and nitrosative stress in a 1-methyl-4-phenyl-1,2,3,6-tetrahydropyridine (MPTP)-induced animal model of Parkinson’s disease (PD).

**Materials and Methods:**

PD model was developed by 4 intraperitoneal (i.p.) injections of MPTP (20 mg/kg) with 2 h intervals in mice. Experimental groups received once daily injection of saline as vehicle (control group) or arbutin (50 mg/kg, i.p.) one week before MPTP injections and this protocol was continued seven days post lesion. Behavioral deficits were evaluated using locomotion test, hanging wire test and forepaw stride length. Parameters indicating the oxidation levels including lipid peroxidation marker (TBARS), nitrite, protein carbonyl levels and antioxidant activity including ferric reducing antioxidant power (FRAP) were assessed in serum and midbrain samples.

**Results:**

Treatment with arbutin improved motor functions in an MPTP-induced PD model compared to control group (p<0.001). Mice treated with MPTP showed reduced levels of FRAP (p<0.001) and increased levels of TBARS (p<0.001), nitrite (p<0.001) and protein carbonyl (p<0.01), compared to the control group. In contrast to the MPTP group, arbutin treatment decreased the levels of TBARS (p<0.05), nitrite (p<0.05), protein carbonyl (p<0.05), and increased FRAP levels (p<0.05) in mice with PD.

**Conclusion:**

These findings suggest that arbutin attenuates the behavioral impairment and oxidative stress in a PD animal model.

## Introduction

Parkinson’s disease (PD) is considered the second most common neurodegenerative disorder after Alzheimer’s disease, affecting approximately 1% of individuals older than 65 years (Eriksen and Petrucelli, 2004 ▶; Singh et al., 2007 ▶). It is associated with progressive degeneration of dopaminergic (DAergic) neurons in the substantia nigra pars compacta (SNpc) region of the brain. Insufficient DA levels result in specific neurological symptoms including tremors, muscle rigidity, slow movements or bradykinesia and postural instability (Wakamatsu et al., 2008 ▶; Dawson et al., 2010 ▶). The molecular mechanisms involved in PD are still unclear; however, numerous studies have implicated that oxidative stress, mitochondrial dysfunction and apoptosis play important roles in pathophysiology of PD (Lin and Beal, 2006 ▶; Levy et al., 2009 ▶). There is general consensus that free radicals are involved in PD (Lin and Beal, 2006 ▶). These radicals can cause damage to macromolecules, including oxidation and single-strand breakage in DNA, lipid peroxidation and formation of protein carbonyls (Giasson et al., 2002 ▶). Reactive oxygen species (ROS) and peroxynitrite degrade polyunsaturated lipids into malondialdehyde (MDA). Thus, thiobarbituric acid reactive substance (TBARS), as a marker of lipid peroxidation may increase in the brain of Parkinsonian patients (Mena et al., 2005 ▶; Sharma et al., 2008 ▶). 

Animal models of PD have been developed to investigate molecular mechanisms and possible therapeutic targets of new drugs (Betarbet et al., 2002 ▶). It has been well documented that administration of 1-methyl-4-phenyl-1,2,3,6-tetrahydropyridine (MPTP) causes physiological symptoms similar to those observed in patients with PD. It has been shown that MPTP inhibits mitochondrial complex I and increases production of ROS, especially superoxide (Gerlach et al., 1991 ▶). 

Current PD treatement approaches include dopamine replacement with L-Dopa, administration of dopaminergic agonists and monoamine oxidase B inhibitors and use of drug combinations that improve motor deficits without reducing the degeneration of DAergic neurons. However, these strategies lose effectiveness over time (Suchowersky, 2002 ▶) and cause side effects such as nausea, vomiting, headache, fatigue, fainting, increased thirst and tremors. Thus, development of new therapeutic approaches for treatment of PD has attracted special attention in recent years (Koppula et al., 2012 ▶). 

Recently, application of natural compounds have received significant attention, and several lines of evidence showed the beneficial effects of ROS scavengers, transition metal (e.g., iron and copper) chelators, non-vitamin natural antioxidant polyphenols and bioenergetic drugs as monotherapy or as parts of an antioxidant cocktail formulation (Mandel and Youdim, 2004 ▶). Interestingly, it has been shown that medicinal herbs such as *Cyperus rotundus* L. (Lee et al., 2010 ▶), *Senna obtusifolia *(L.) H.S. Irwin & Barneby (Ju et al., 2010a ▶) and *Platycladus orientalis *(L.) Franco (Ju et al., 2010b ▶) exert protective effects against neurotoxicity in neurodegenerative disease models. 

Arbutin is a glycosylated hydroquinone that is naturally found in various plant species such as leaves of bearberry (Ericaceae), pear trees (Rosaceae) and *Bergenia crassifolia* (Saxifragaceae) (Carmen et al., 2009 ▶). *In vitro* and *in vivo* experiments have demonstrated that arbutin is effective against inflammation of the bladder, high blood pressure and urinary stones (Shahaboddin et al., 2011 ▶; Yousefi et al., 2013 ▶). Additionally, arbutin also induces anti-inflammatory (Lee and Kim, 2012 ▶), antioxidant, free radical-scavenging (Myagmar et al., 2004 ▶; Khadir et al., 2015 ▶), antihyperglycemic, antihyperlipidemic, and bactericidal effects (Petkou et al., 2002 ▶; Shahaboddin et al., 2011 ▶). 

To the best of our knowledge, the possible protective effect of arbutin against PD has not been previously reported. This study was designed to evaluate the effect of arbutin on behavioral impairments in an MPTP-induced model. Furthermore, the levels of lipid peroxidation marker (TBARS), nitrite, protein carbonyl levels and total antioxidant capacity were assessed in animals receiving arbutin.

## Materials and Methods

**Chemicals**

MPTP-HCl, thiobarbituric acid (TBA) and arbutin were obtained from Sigma-Aldrich (USA). Malondialdehyde (MDA), nitric oxide, and protein carbonyl assay kits were purchased from ZellBio GmbH (Germany). 2,4,6-Tris (2-pyridyl)-s-triazine (TPTZ) was obtained from Merck company (Germany). Arbutin and MPTP were dissolved in sterile saline and their appropriate doses were selected according to previous reports (Khadir et al., 2015 ▶; Essawy et al., 2017 ▶).

**Animals**

In this study, 21 male albino mice (NMRI) weighing 30-35 g were used. All experimental procedures were approved by the Ethics Committee of Babol University of Medical Sciences which was in accordance with international guideline for use and care of laboratory animals. 

**Experimental design**

 Animals were randomly divided into 3 experimental groups (n=7) as follows: 

Group 1: Control group which received i.p. injection of saline. 

Group 2: saline+MPTP: in this group, saline, as arbutin vehicle, was given i.p. for 7 days. From the 8th day, animals received MPTP injections (4 i.p. injections of MPTP (20 mg/kg) with 2-hr intervals) (Essawy et al., 2017 ▶). Administration of saline was continued 1 week post MPTP injections. 

Group 3: animals received arbutin (50 mg/kg, i.p.) for 7 days and experimental procedure was the same as that mentioned for group 2. Arbutin was administrated 2 hr before the first MPTP injection. 

On the 14th day of the experiment, behavioral studies were performed to evaluate motor skill abnormalities. After that, serum and midbrain tissues were collected for biochemical assessment.

**Assessment of motor function**

*Locomotor test*

General locomotor activity was examined in a plastic box (40×40×30 cm) for 15 min. Locomotion was automatically measured by a digital counter that recorded photocell beam interruptions (Moreira et al., 2010 ▶; Luo et al., 2011 ▶). 

*Hanging wire test*

Mice were placed on a horizontal grid and supported until they held the grid. The grid was then inverted and the mice were allowed to hang upside down for 30 sec; 10 chances were given with 1-min intervals, and the number of falls was recorded. The percentage of success was measured as maximum time hanging in 30 sec×100 (Mohanasundari et al., 2006 ▶).

*Forepaw stride length during walking*

Animals had their forepaws placed in black ink and the length of forepaw steps was measured during normal walking across a clean sheet of paper. Stride length was calculated from the mid-digit toe of the first step to the heel of the second step. The distance between steps was measured on the same side of the body (Tillerson et al., 2002 ▶).

**Biochemical assays**

*Tissue preparation*

Blood was collected and separated serum was stored at -20°C for further analysis. Animals were then sacrificed and brain tissues were quickly dissected out and kept on ice. The midbrains were homogenized using an ultra-TURRAX homogenizer (IKA T25 digital, Germany) at 3,000 rpm in cold normal saline to obtain a 10% homogenate solution. The homogenate was centrifuged at 2,000 rpm for 10 min at 4°C. Then, the supernatant was collected and aliquots were stored at -80°C.

*Determination of total antioxidant activity*

Total antioxidant activity was estimated by a ferric reducing antioxidant power (FRAP) assay as described previously (Khadir et al., 2015 ▶). This method is based on the reduction of a ferric tripyridyltriazine complex to its ferrous, colored form in the presence of antioxidants. A FRAP reagent, containing TPTZ solution (10 mM) in HCl (40 mM), FeCl_3_ (20 mM), and acetate buffer (0.3 M) at pH 3.6 was used; the reagent was freshly prepared. The FRAP assay was performed according to a standard method. The same volumes of each sample and standards (50 μl each) were added to 1.5 ml of FRAP reagent. After 10 min incubation at 37°C, absorbance was measured at 593 nm. Different concentrations of FeSO_4_ (125-1,000 μM) were used as standard solutions (Benzie and Strain, 1996 ▶).

*Nitric oxide assay*

Nitric oxide levels were measured based on manufacturer's protocol (ZellBio GmbH, Germany). In brief, using this kit, the NO content was indirectly assessed by measuring the concentrations of nitrates and nitrites, which can react with a chromogenic agent to produce a pink azo compound. Absorbance of the compound was measured at 540 nm. Nitrite levels were calculated in unknown samples based on a standard curve (Khanjarsim et al., 2017 ▶). 

*Malondialdehyde assay*

The MDA assay kit uses an adduct formed by the reaction between MDA and TBA under high temperature. The level of MDA was measured in an acidic medium under heat (100°C), using a colorimetric (535 nm) method. The linear regression equation for a standard curve was calculated according to the concentration of the standard and the optical density (OD) value. This linear equation was used to calculate the concentration of samples (Khadir et al., 2015 ▶). 

*Protein carbonyl assay*

The protein carbonyl enzyme-linked immunosorbent assay is based on biotin double-antibody sandwich technology. The absorbance (OD) of samples was measured at 450 nm. The absorbance of the solution and the concentration of human protein carbonyl are positively correlated. Total protein concentration of serum and brain homogenates was determined using Bradford method.

**Statistical analysis**

Data were expressed as mean±SEM values. Statistical analysis was performed using SPSS software version 18.0. One-way analysis of variance followed by Tukey’s *post hoc* test was used for data analysis. P values less than 0.05 were considered statistically significant.

## Results

**Effect of arbutin on behavioral deficit in an MPTP-induced animal model**

**Figure 1:**
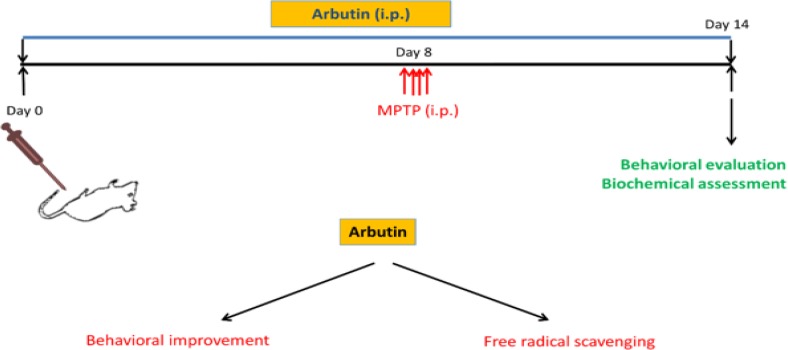
Schematic representation of the present experiments

**Figure 2:**
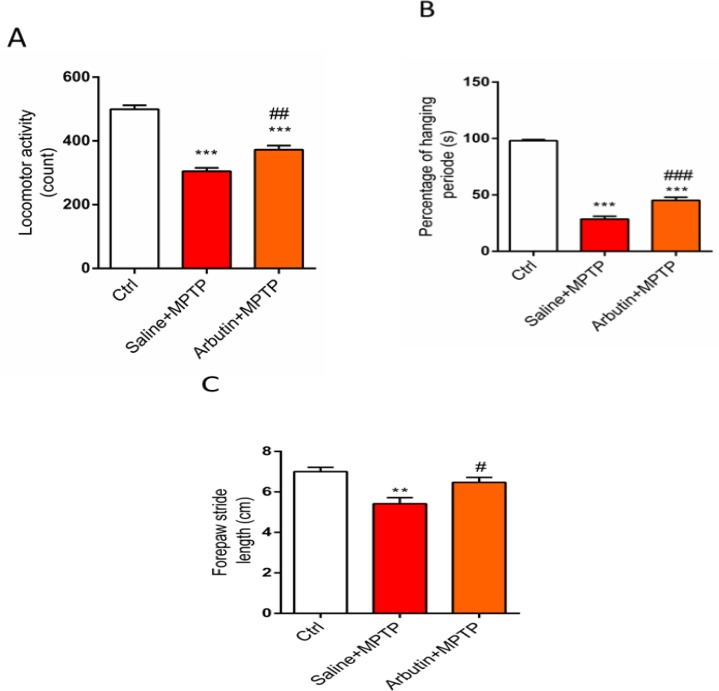
Comparison of locomotor activity (A), hanging time (B) and forepaw stride length (C) among control, MPTP, and arbutin+MPTP groups. Values are expressed as mean±SEM^. **^p<0.01 and ^***^p<0.001 show significant differences as compared to control; ^#^p<0.05, ^##^p<0.01 and ^###^p<0.001 show significant differences as compared to the MPTP group (n=7)

In order to determine the effect of arbutin administration on MPTP-induced behavioral impairment, motor activity was evaluated and compared among experimental groups. Figure 1 shows a schematic timeline of the experiments. In the MPTP group, the motor activity was significantly decreased compared to the control group (p<0.001). A significant reduction in motor activity was also found in animals treated with arbutin (p<0.001). Additionally, mice treated with arbutin exhibited more activity than mice received MPTP alone (p<0.01) (Figure 2A). 

The percentage of hanging period was also assessed in animal groups. Our data showed a significant reduction in the hanging time of MPTP receiving animals compared to the control group (p<0.001) (Figure 2B). The hanging time was decreased in arbutin-treated animals compared to control group (p<0.001). Interestingly, arbutin treatment could significantly increase the percentage of hanging period compared to the saline+MPTP receiving animals (p<0.001) (Figure 2B). 

Furthermore, the forepaw step distance was also evaluated in animals and our results indicated that administration of MPTP significantly reduced this parameter compared to the control group (p<0.01). Mice that received arbutin showed a significant increase in forepaw step distance compared to the saline+MPTP group (p<0.05) (Figure 2C).

**Effect of arbutin on oxidative stress levels in an MPTP-induced animal model**

**Figure 3:**
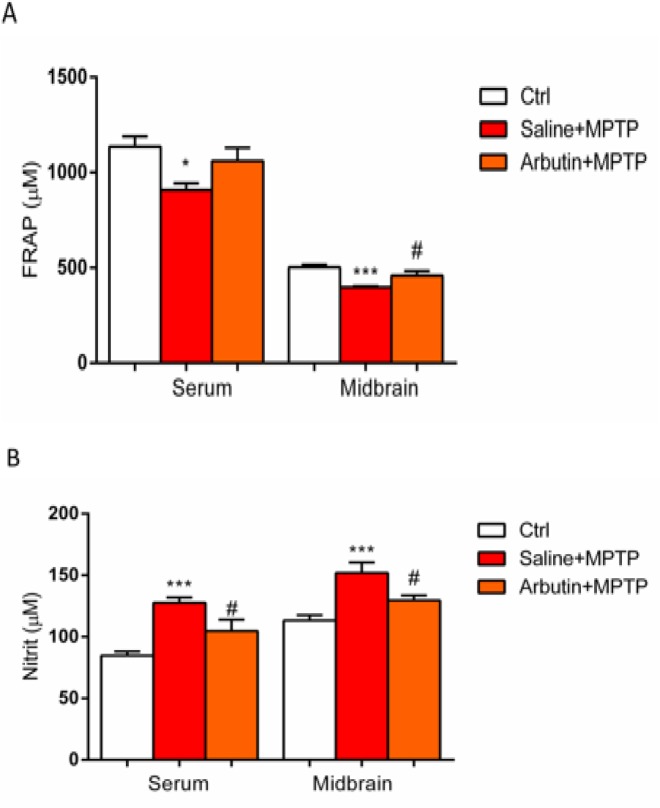
Measurement of total antioxidant activity (A) and nitrite levels (B) in serum and midbrain samples. Values are expressed as mean±SEM. ^*^p<0.05 and ^***^p<0.001 show significant differences as compared to control; ^#^p<0.05 shows significant differences as compared to the MPTP group (n=7).

The effect of arbutin treatment on FRAP, MDA, nitrite, and protein carbonyl levels were evaluated in serum and midbrain tissues of animals. In animals that received MPTP, a reduction was observed in serum levels of FRAP (p<0.05) and midbrain tissues (p<0.001) compared to the control group. Interestingly, our data demonstrated that the FRAP levels were increased in the brain tissues of arbutin-treated PD mice compared to the saline+MPTP group (p<0.05). Although, in comparison with the MPTP group, the levels of FRAP were increased in serum samples of arbutin-treated animals, but this effect was not statistically significant (Figure 3A). 

In addition, a significant increase in nitrite levels was found in the MPTP group compared to the control group (p<0.001) and treatment of animals with arbutin significantly reduced the levels of nitrite in the brain (p<0.05) and serum (p<0.05) of MPTP receiving animals (Figure 3B).

**Figure 4:**
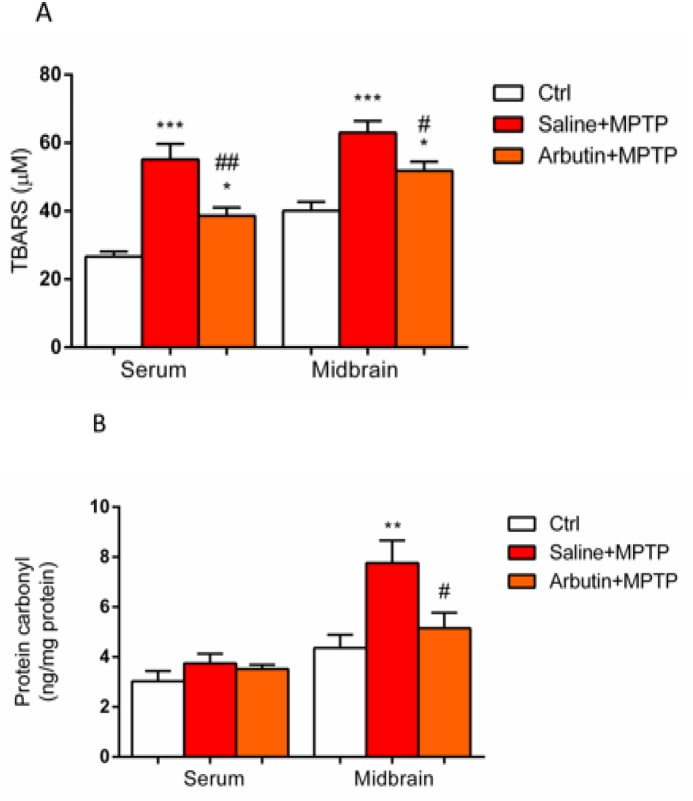
Measurement of TBARS (A) and protein carbonyl levels (B) in serum and midbrain. Values are expressed as mean±SEM. ^*^p<0.05, ^**^p<0.01 and ^***^p<0.001 show significant differences as compared to control; ^#^p<0.05 and ^##^p<0.01 show significant differences as compared to the MPTP group (n=7).

 Furthermore, TBARS level, as a marker of lipid peroxidation, was found to be elevated in both brain and serum samples of the MPTP group compared to the control group (p<0.001). A significant difference in TBARS level was also observed between control and arbutin receiving animals (p<0.05). Our data demonstrated that treatment with arbutin significantly attenuated the level of TBARS in the brain (p<0.01) and serum (p<0.05) of MPTP receiving mice (Figure 4A). 

Protein carbonyl assessment indicated that its level significantly increases in the brain of the MPTP group compared to the control group (p<0.01) and arbutin treatment reduced the levels of protein carbonyl in brain tissues of MPTP receiving animals (p<0.05) (Figure 4B). We could not observe any significant difference in protein carbonyl levels of serum samples between saline+MPTP and arbutin+MPTP treated animals (Figure 3B).

## Discussion

In the present study, arbutin improved the behavioral performance including locomotor activity, hanging time and forepaw stride length. Additionally, arbutin treatment reduced the MPTP-induced oxidative stress and increased total antioxidant capacity in arbutin-treated animals. 

It has been suggested that oxidative stress and mitochondrial dysfunction have important roles in PD pathogenesis (Przedborski et al., 2004 ▶; Levy et al., 2009 ▶). Animals treated with MPTP showed behavioral, neurochemical and histological changes similar to those observed in humans with PD (Mena et al., 2005 ▶; Wakamatsu et al., 2008 ▶). In line with these findings, we also observed a reduction in locomotor activity of MPTP-treated mice. Motor dysfunction is induced by the progressive loss of dopaminergic neurons of the substantia nigra region of the brain (Dawson et al., 2010 ▶). Interestingly, compared with MPTP receiving animals, administration of arbutin improved motor functions in this animal model of PD. Moreover, the hanging time and forepaw step distance were significantly decreased in the MPTP group. Our findings are supported by the results of previous studies that evaluated these behavioral parameters (Tillerson et al., 2002 ▶; Luo et al., 2011 ▶; Essawy et al., 2017 ▶). 

To find the possible protective mechanism(s) of arbutin, the levels of oxidation were evaluated in experimental groups. In the current study, MPTP receiving mice showed a reduction in total antioxidant activity in serum and midbrain tissues. It has been shown that MPTP is rapidly metabolized to the toxic ion 1-methyl-4-phenyl-piperidinium (MPP+) in the brain (Langston et al., 1984 ▶). Accumulation of MPP+ in the mitochondria of dopaminergic neurons inhibits complex-I of the electron transport chain, causes severe depletion of ATP and increases the production of ROS which leads to the loss of dopaminergic neurons (Przedborski et al., 2004 ▶; Essawy et al., 2017 ▶). In the present study, arbutin increased FRAPS levels in the brains of PD mice. Previous studies have demonstrated the antioxidative and free radical-scavenging properties of arbutin, and proved that arbutin is more effective at the dose of 50 mg/kg (Khadir et al., 2015 ▶). Furthermore, our data showed that arbutin causes a significant reduction in nitrite levels following MPTP injections. 

Nitric oxide, produced by nitric oxide synthase, participates in a cascade of events leading to the degeneration of dopaminergic neurons (Liberatore et al., 1999 ▶). Reactive nitrogen species such as peroxynitrite are probably involved in MPTP toxicity (Przedborski and Vila, 2001 ▶). Peroxynitrite can cause damage to macromolecules through DNA oxidation, lipid peroxidation and formation of protein carbonyls (Giasson et al., 2002 ▶). Our findings also showed elevated levels of a marker of lipid peroxidation (TBARS) in the brain and serum of PD mice. An increased amount of TBARS was also reported in the brains of MPTP-treated monkeys (Marzatico et al., 1993 ▶) and mice (Sankar et al., 2007 ▶). Reduced levels of TBARS in the arbutin-treated PD mice showed that arbutin probably removes free radicals and inhibits lipid peroxidation. In consistence with our data, treatment of PD mice with different kinds of antioxidants significantly reduced the levels of TBARS in the brain tissues (RajaSankar et al., 2009 ▶). Additionally, it has also been shown that other polyphenols such as curcumin or resveratrol can effectively reduce the behavioral impairment, oxidative stress and neuronal loss following MPTP administration (Rajeswari, 2006 ▶; Lu et al., 2008 ▶; He et al., 2015 ▶). It has been suggested that the neuroprotective effects of these polyphenols are partly mediated through free-radical scavenging (Lu et al., 2008 ▶). 

Our study also indicated that arbutin can decrease the formation of protein carbonyl in the brains of PD mice. Protein carbonyls, among various oxidative modifications of amino acids, are regarded as early markers of protein oxidation (Dalle-Donne et al., 2003 ▶). The accumulation of oxidized protein reflects the rate of protein oxidation and oxidized protein degradation (Berlett and Stadtman, 1997 ▶). 

In conclusion, the results of this study showed that arbutin can effectively attenuate behavioral deficits and reduce oxidative and nitrosative stress in MPTP-induced PD model. However, further studies are needed to clarify the exact molecular mechanisms by which arbutin can protect dopaminergic neurons in MPTP receiving animals.
